# Effect of Primary Versus Revisional One Anastomosis Gastric Bypass (OAGB) on Fatty Acid Profile

**DOI:** 10.1007/s11695-023-06928-1

**Published:** 2023-11-15

**Authors:** Michal Szymanski, Maciej Wilczynski, Alicja Pakiet, Lukasz Kaska, Monika Proczko-Stepaniak, Justyna Bigda, Tomasz Sledzinski, Adriana Mika

**Affiliations:** 1 Department of General, Endocrine, and Transplant Surgery, Medical University of Gdansk, ul. Smoluchowskiego 17, 80-214 Gdansk, Poland; 2 Department of Environmental Analysis, University of Gdansk, Wita Stwosza 63, 80-308 Gdansk, Poland; 3 Department of Pharmaceutical Biochemistry, Medical University of Gdansk, ul. Debinki 1, 80-211 Gdansk, Poland

**Keywords:** Obesity, OAGB, Revisional bariatric surgery, Fatty acids

## Abstract

**Introduction:**

One anastomosis gastric bypass (OAGB) is one option of a revisional procedure for failed sleeve gastrectomy. Moreover, it can be used as a primary bariatric procedure, and is an effective surgery resulting in significant weight loss and the resolution or improvement of obesity-associated medical problems, accompanied by low perioperative complications. However, as with any therapy, OAGB has its limitations, including micronutrient deficiency or malnutrition. In our study, we compared the fatty acid (FA) profile in serum of patients after both primary OAGB (pOAGB) and revisional OAGB (rOAGB) to identify potential postsurgical FA alterations.

**Methods:**

This is a retrospective study on patients with obesity who underwent OAGB procedures (pOAGB *n*=68; rOAGB *n*=17), conducted from 2016 to 2018. In blood, we analyzed a series of biochemical parameters, and in the serum, the FA profile was determined using gas chromatography-mass spectrometry.

**Results:**

The percentage of excess BMI loss (% EBMIL) after pOAGB was 73.5 ± 2.47% in comparison to 45.9 ± 4.15% in the rOAGB group (*p*<0.001). In contrast to the lack of effect of rOAGB on most polyunsaturated FAs, in the pOAGB group, there was a decrease in eicosapentaenoic acid, and eicosatetraenoic and docosahexaenoic acid levels (*p*<0.001). We also found a decrease in very long-chain FAs (VLCFAs) and an increase in branched-chain FAs (BCFAs) after both types of OAGB procedure.

**Conclusions:**

Both OAGB procedures improved the profile of most FAs, leading to a decrease in VLCFAs, which are considered harmful, and an improvement in BCFAs, which are considered to be beneficial. There is a need to further investigate the possibility of n-3 polyunsaturated FA supplementation after pOAGB, due to the large decrease in these FAs after pOAGB.

**Graphical Abstract:**

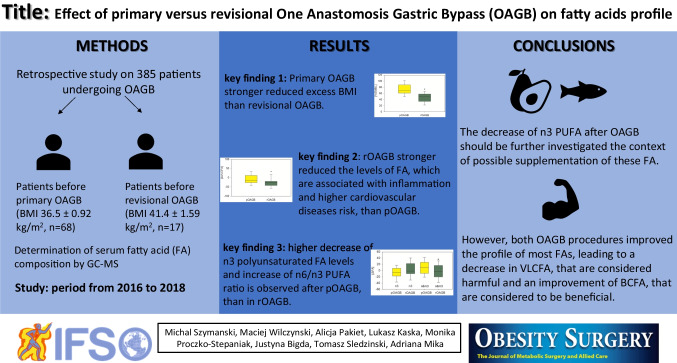

## Introduction

Bariatric surgery (BS) is currently the option of choice for patients living with severe obesity and obesity-related diseases. Laparoscopic sleeve gastrectomy (SG) is the most common bariatric procedure performed worldwide [1]. Several studies with a long-term follow-up focused on the high rate of revisions following SG [2, 3]. Conversion is performed mostly because of insufficient weight loss (IWL), weight regain (WR), and symptomatic gastroesophageal reflux disease (GERD) [2, 3]. Therefore, there is an ongoing debate regarding revisional surgical procedures following SG. Currently, one of the main options is Roux-en-Y gastric bypass (RYGB) interestingly [4]. The second surgical conversion option is often one anastomosis gastric bypass (OAGB) [5]. There is a higher rate of complications after revisional BS than in the primary operation [6]. Surgical intervention for internal herniation was more prevalent in the RYGB group, whereas surgical intervention for biliary reflux was prevalent in the OAGB group [7]. However, after OAGB, an improvement in T2DM was reported in almost 90% of patients [8]. Another study indicates that OAGB surgery as a revisional procedure for failed SG is an effective bariatric surgical procedure producing significant weight loss and the resolution or improvement of associated medical problems, accompanied by low perioperative complications [7]. This problem was summarized perfectly by Brethauer et al. [9], who wrote that severe obesity is a chronic disease and acceptable long-term management after a primary bariatric procedure should include the surgical options of conversion, correction, or another adjuvant therapy to achieve an acceptable treatment effect. To sum up, it is important to notice that the qualification criteria for pOAGB and rOAGB differ. Patients qualified for rOAGB are after SG, and although they experienced WR, some metabolic improvements are present [7, 10].

It is well documented in the literature that bariatric surgery causes not only weight reduction, but also improves metabolic parameters, metabolites [11], and among them, some fatty acids (FAs) [12]. The effects of OAGB and other types of BS, such as RYGB and SG, on the levels of bile acids (BAs) and free fatty acids (FFAs) were compared in our previous papers [13, 14]. The abovementioned changes in the examined bioactive compounds may contribute to a great improvement in glucose metabolism and insulin sensitivity after surgery. None of the studies described changes of FA and its potential deficiencies, in a group of patients following revisional BS. Therefore, the aim of the study was to compare the effect of primary OAGB (pOAGB) and revisional OAGB (rOAGB) on FA profiles and identify potential postsurgical FA deficiencies.

## Methods

### Patients

This is a retrospective study based on a chart review of 385 patients operated on at the Department of General, Endocrine and Transplant Surgery at the Medical University of Gdansk, between 2016 and 2018. In our institution, OAGB is the first choice bypass surgery, because of the better improvement in T2DM than with RYGB [15]. The inclusion criteria for pOAGB were in line with the guidelines of the International Federation for the Surgery of Obesity (IFSO), the International Federation for the Surgery of Obesity – European Chapter (IFSO-EC), the European Association for the Study of Obesity (EASO) [16] and in accordance with the recommendations of the Bariatric Chapter of the Association of Polish Surgeons [17], and included: BMI > 35 with co-morbidity/metabolic disease or BMI > 40 without diseases; age over 18 years; without significant gastrointestinal diseases (e.g., enteritis such as Crohn’s disease); without acute mental disorders with psychotic symptoms (e.g., schizophrenia). The exclusion criteria are the opposite of those mentioned above, together with pregnancy, active autoimmune, inflammatory or infectious diseases, untreated viral hepatitis, current oncological treatment, known alcohol consumption (>20 g/day), or kidney diseases [16]. The inclusion criteria for rOAGB were IWL (defined as <50% EWL), WR (defined as ≥ 20% weight regain of the weight lost) and the lack of remission of obesity-associated diseases or recurrence of diseases associated with obesity after SG [18, 19]. The exclusion criteria for both pOAGB and rOAGB were confirmed Barrett’s esophagus or severe esophagitis (Los Angeles classification C or D) [20]. Both pOAGB and RYGB as well as other bypass surgeries have a beneficial impact regarding an improvement in T2DM [21]. In our institution, pOAGB is offered for patients with T2DM, based on our previous study, which proves a better resolution of T2DM after OAGB than after RYGB [15]. Revisional OAGB is offered as a revisional procedure mostly because of WR or poor weight loss [5, 22].

### Blood Samples

Blood was obtained from all subjects before OAGB (rOAGB and pOAGB) and 6–9 months after rOAGB/pOAGB. Blood samples were collected in the morning from all study subjects. The biochemical and anthropometric characteristics of patients with obesity are presented in Table 1, whereas the postsurgical changes of these parameters in both groups are presented in Table 2.

**Table 1:** Selected biochemical and anthropometric characteristics in the study groups

	pre pOAGB	Post pOAGB	Pre rOAGB	post rOAGB	*p*-value (pre vs post pOAGB)	*p*-value (pre vs post rOAGB)	*p-*value pre pOAGB vs pre rOAGB
Age (years)	49.2 ± 1.39		51.3 ± 2.59		NT	NT	0.734
Sex (female %)	52% (76.5%)		13 (76.5%)				
BMI (kg/m2)	36.5 ± 0.92	27.9 ± 0.63	41.4 ± 1.59	33.8 ± 1.15	**<0.001**	**<0.001**	**<0.008**
CRP-hs (mg/L)	2.70 ± 0.17	1.96 ± 0.16	2.13 ± 0.30	1.96 ± 0.37	<0.001*	0.426*	0.208*
LDL (mg/dL)	126 ± 4.06	125 ± 4.21	141 ± 14.4	133 ± 13.8	0.437*	0.359*	0.727*
HDL (mg/dL)	42.2 ± 1.27	51.1 ± 1.49	40.3 ± 3.50	54.0 ± 4.21	**<0.001**	**<0.001**	0.413*
CHOL (mg/dL)	185 ± 4.67	203 ± 5.62	210 ± 17.1	217 ± 18.1	0.037*	0.607	0.316*
TG (mg/dL)	156 ± 8.57	129 ± 6.20	109 ± 15.0	96 ± 9.27	0.007*	0.164*	0.507*
GLU (mg/dL)	141 ± 5.19	105 ± 2.76	146 ± 14.8	119 ± 9.39	<0.001*	0.126	0.941*
ALB (g/L)	37.0 ± 0.49	41.5 ± 0.57	36.7 ± 0.92	43.9 ± 1.51	**<0.001**	0.004*	0.575*
PRO (g/L)	62.0 ± 1.16	66.7 ± 0.83	64.9 ± 4.48	71.0 ± 3.20	<0.001*	0.217	0.919*

*BMI* body mass index, *CRP-hs* high-sensitive C-reactive protein, *LDL* low-density lipoprotein, *HDL* high-density lipoprotein, *CHOL* total cholesterol, *TG* triacylglycerols, *GLU* glucose, *ALB* total albumin level in serum, *PRO* total protein level in serum, *OAGB* one anastomosis gastric bypass. *p for non-parametric tests, *NT* not tested

Boldface-statistically significant value

**Table 2:** Changes of selected biochemical and anthropometric characteristics in the study

	pOAGB	rOAGB	*p-*value
%EMBIL	73.5 ± 2.47%	45.9 ± 4.15%	**<0.001**
CRP-hs (mg/L)	−17.5 ± 6.52	4.33 ± 24.2	0.332*
LDL (mg/dL)	4.06 ± 4.84	−1.77 ± 12.0	0.406*
HDL (mg/dL)	24.4 ± 3.96	37.3 ± 6.68	0.149
CHOL (mg/dL)	13.8 ± 4.32	6.36 ± 9.53	0.603*
TG (mg/dL)	−8.03 ± 4.91	0.99 ± 17.4	0.986*
GLU (mg/dL)	−21.7 ± 2.91	−14.8 ± 7.37	0.522
ALB (g/L)	12.9 ± 1.81	20.7 ± 6.10	0.214
PRO (g/L)	8.98 ± 1.89	13.5 ± 9.55	0.959*

%*EMBIL*, percentage of the excess BMI loss; *CRP*-*hs*, high-sensitive C-reactive protein; *LDL*, low-density lipoprotein; *HDL*, high-density lipoprotein; *CHOL*, total cholesterol; *TG*, triacylglycerols; *GLU*, glucose; *ALB*, total albumin level in serum; *PRO*, total protein level in serum; *OAGB*, one anastomosis gastric bypass. *p for non-parametric tests

Boldface-statistically significant value

### Analysis of Fatty Acids

The preparation of FAs from serum included the extraction of total lipids using the method described by Folch et al. [23] with a chloroform-methanol mixture (2:1, v/v) and the hydrolysis of extracted lipids using KOH-methanol (potassium hydroxide solution in methanol). 10% boron trifluoride in methanol solution was used to obtain FA methyl esters (FAMEs) and a gas chromatography-mass spectrometry (GC-MS) analysis of FAMEs was conducted with a GC-MS QP-2010 SE (Shimadzu, Kyoto, Japan), as described previously [12].

### Statistical Analysis

Data are expressed as the mean value ± the standard error of the mean (SEM). The *p*-value was considered significant at < 0.05. The data analysis was performed in SigmaPlot 14.5 (Systat Software Inc., San Jose, CA, USA). Comparisons between patients were made with the Student’s *t-*test (for parametric data) and the Mann-Whitney Rank Sum Test (for non-parametric data). These tests were preceded by an analysis of variance to select the appropriate type of test. Comparisons between patients before and after surgery were made with the paired Student’s t-test (for parametric data) and the Wilcoxon signed-rank test (for non-parametric data). Correlations between pairs of variables were determined on the basis of linear regression analysis (Pearson correlation coefficient).

## Results

The study groups consist of 68 patients who underwent pOAGB (mean age 49.2 ± 1.39 years; 16 males, 52 females) and 17 patients who underwent rOAGB (mean age 51.3 ± 2.59 years; 4 males, 13 females) due to failed SG. 300 patients were excluded from the study based on our exclusion criteria. The mean time between SG and rOAGB was 16.4 ± 1.42 months.

Patients qualified for pOAGB were characterized by a lower BMI than the group qualified for rOAGB (36.5kg/m^2^ ± 0.92 vs 42.9 kg/m^2^ ± 1.23; *p*<0.001) (Table 1). The BMI loss after surgery (%EBMIL) in these patients was 73.5 ± 2.47% in comparison to 45.9 ± 4.15% in the rOAGB group (*p*<0.01) (Table 2). After pOAGB, almost all parameters were improved, whereas after rOAGB, significant changes were observed only in BMI, HDL, and albumin (Table 1).

Table 3 indicates several changes in FA profiles in both variants of the OAGB procedure. Changes in FA levels were observed in 38 of all 56 FAs after pOAGB. However, an analysis of FAs in rOAGB showed changes in only 1/3 of all FAs after this variant of surgery. The biggest differences after pOAGB, which were not observed after rOAGB, concerned FAs which are considered pro-healthy, including polyunsaturated fatty acids (PUFAs) and branched-chain fatty acids (BCFAs). On the other hand, very long-chain fatty acids (VLCFAs; with C≥22atoms), which are associated with a higher risk of cardiovascular diseases, were reduced after rOAGB and after pOAGB, compared with before rOAGB and pOAGB (Table 3).

**Table 3:** Fatty acid content (%) in patients with obesity serum. Values are mean ± SEM

	Pre pOAGB	Post pOAGB	Pre rOAGB	Post rOAGB	*p-*value (pre vs post pOAGB)	*p-*value (pre vs post rOAGB)	*p-*value pre (pOAGB vs rOAGB)
C10:0	0.036 ± 0.007	0.010 ± 0.001	0.024 ± 0.009	0.008 ± 0.001	<0.001*	<0.001*	0.669*
C12:0	0.10 ± 0.004	0.19 ± 0.019	0.09 ± 0.009	0.12 ± 0.037	<0.001*	0.670*	0.548*
C14:0	0.85 ± 0.025	1.23 ± 0.057	0.83 ± 0.094	1.04 ± 0.121	<0.001*	0.024	0.183*
C16:0	24.5 ± 0.15	23.9 ± 0.22	24.9 ± 0.45	24.7 ± 0.57	<0.001*	0.464	0.685*
C18:0	6.03 ± 0.05	6.42 ± 0.08	6.48 ± 0.15	6.68 ± 0.15	<0.001*	0.093	0.022
C20:0	0.10 ± 0.003	0.11 ± 0.004	0.08 ± 0.005	0.07 ± 0.004	0.046	0.385	0.001*
C22:0	0.17 ± 0.006	0.14 ± 0.006	0.14 ± 0.009	0.11 ± 0.007	<0.001*	0.008*	0.217*
C24:0	0.13 ± 0.005	0.12 ± 0.007	0.10 ± 0.007	0.07 ± 0.006	0.004*	0.001	0.019*
**ECFA**	**31.9 ± 0.14**	**32.1 ± 0.26**	**32.7 ± 0.53**	**32.8 ± 0.64**	0.919*	**0.786**	0.323*
C11:0	0.005 ± 0.000	0.005 ± 0.000	0.007 ± 0.001	0.006 ± 0.001	0.102*	0.313*	0.066*
C13:0	0.014 ± 0.001	0.016 ± 0.001	0.016 ± 0.002	0.013 ± 0.001	0.050*	0.031*	0.224*
C15:0	0.24 ± 0.007	0.28 ± 0.011	0.22 ± 0.012	0.23 ± 0.015	<0.001*	**0.316**	0.357*
C17:0	0.23 ± 0.004	0.24 ± 0.006	0.23 ± 0.013	0.23 ± 0.012	0.015	0.960	0.916
C19:0	0.018 ± 0.001	0.019 ± 0.001	0.021 ± 0.002	0.022 ± 0.002	0.949*	0.718	0.253*
C21:0	0.017 ± 0.001	0.015 ± 0.002	0.012 ± 0.002	0.010 ± 0.002	0.031*	0.209	0.007*
C23:0	0.060 ± 0.003	0.042 ± 0.002	0.049 ± 0.003	0.030 ± 0.002	<0.001	<0.001	0.076*
**OCFA**	**0.59 ± 0.012**	**0.62 ± 0.018**	**0.56 ± 0.022**	**0.55 ± 0.024**	**0.048**	**0.542**	**0.429**
2,6,10-methyl-12:0	0.011 ± 0.001	0.016 ± 0.002	0.008 ± 0.001	0.011 ± 0.001	<0.001*	0.156*	0.069*
anteiso 12-M-14:0	0.028 ± 0.001	0.045 ± 0.003	0.033 ± 0.004	0.038 ± 0.004	<0.001*	0.067*	0.396*
anteiso 14-M-16:0	0.058 ± 0.003	0.083 ± 0.005	0.063 ± 0.005	0.095 ± 0.009	<0.001*	<0.001	0.346*
anteiso 16-M-18:0	0.029 ± 0.001	0.040 ± 0.002	0.030 ± 0.003	0.039 ± 0.003	<0.001*	0.042*	0.835*
anteiso 20-M-22:0	0.017 ± 0.001	0.020 ± 0.002	0.011 ± 0.001	0.010 ± 0.000	0.033*	0250*	0.040*
**anteiso BCFA**	**0.13 ± 0.005**	**0.19 ± 0.008**	**0.13 ± 0.010**	**0.18 ± 0.012**	<0.001*	**<0.001**	0.548*
iso 12-M-13:0	0.009 ± 0.001	0.010 ± 0.001	0.010 ± 0.002	0.008 ± 0.001	0.059*	0.641*	0.863*
iso 13-M-14:0	0.018 ± 0.001	0.026 ± 0.001	0.022 ± 0.003	0.024 ± 0.003	<0.001*	0.176*	0.360*
iso 14-M-15:0	0.039 ± 0.002	0.054 ± 0.002	0.039 ± 0.002	0.055 ± 0.004	<0.001*	<0.001	0.812*
iso 15-M-16:0	0.068 ± 0.002	0.082 ± 0.004	0.068 ± 0.007	0.090 ± 0.009	<0.001*	0.023	0.810*
iso 20-M-21:0	0.012 ± 0.001	0.012 ± 0.001	0.007 ± 0.001	0.008 ± 0.001	0.375*	0.813*	0.021*
**Iso BCFA**	**0.15 ± 0.005**	**0.18 ± 0.007**	**0.15 ± 0.007**	**0.18 ± 0.014**	**<0.001**	**0.046**	0.463*
**Total BCFA**	**0.29 ± 0.009**	**0.39 ± 0.014**	**0.29 ± 0.013**	**0.38 ± 0.024**	**<0.001**	**<0.001**	0.416*
**Total SFA**	**32.7 ± 0.15**	**33.1 ± 0.26**	**33.5 ± 0.54**	**33.7 ± 0.67**	**0.496**	**0.684**	0.367*
C14:1	0.054 ± 0.003	0.070 ± 0.005	0.052 ± 0.008	0.061 ± 0.012	0.008*	0.384	0.641*
C16:1	3.52 ± 0.11	3.45 ± 0.12	3.08 ± 0.18	3.49 ± 0.22	0.499	0.120*	0.145*
C18:1	30.1 ± 0.32	30.0 ± 0.27	30.7 ± 0.57	31.3 ± 0.85	0.881	0.375	0.397*
C19:1	0.022 ± 0.001	0.024 ± 0.001	0.026 ± 0.002	0.026 ± 0.002	0.214*	0.854	0.062*
C20:1	0.16 ± 0.005	0.16 ± 0.005	0.17 ± 0.009	0.13 ± 0.007	0.656*	<0.001	0.297*
C22:1	0.020 ± 0.001	0.020 ± 0.001	0.016 ± 0.001	0.014 ± 0.001	0.582*	0.301*	0.305*
C24:1	0.30 ± 0.013	0.37 ± 0.012	0.20 ± 0.020	0.29 ± 0.025	<0.001	<0.001*	0.001
**Total MUFA**	**34.2 ± 0.36**	**34.2 ± 0.32**	**34.2 ± 0.64**	**35.3 ± 0.97**	**0.952**	**0.190**	0.982*
CPOA2H	0.16 ± 0.004	0.16 ± 0.007	0.16 ± 0.010	0.15 ± 0.009	0.373*	0719	0.878*
ALA	0.23 ± 0.010	0.23 ± 0.013	0.19 ± 0.020	0.21 ± 0.017	0.707	0.292	0.107*
EPA	0.75 ± 0.07	0.59 ± 0.02	0.73 ± 0.09	0.72 ± 0.07	0.078*	0.935	0.697*
ETA	0.05 ± 0.002	0.04 ± 0.002	0.04 ± 0.003	0.04 ± 0.003	<0.001*	0.221	0.037*
DHA	1.32 ± 0.052	1.13 ± 0.038	1.16 ± 0.072	1.11 ± 0.078	<0.001*	0.610	0.212*
DPAn-3	0.32 ± 0.008	0.36 ± 0.011	0.26 ± 0.017	0.30 ± 0.021	<0.001	0.018*	0.008
**Total PUFAn-3**	**2.67 ± 0.12**	**2.36 ± 0.06**	**2.38 ± 0.16**	**2.38 ± 0.15**	0.008*	**0.997**	0.115*
LA	22.6 ± 0.33	22.9 ± 0.40	22.8 ± 1.03	21.8 ± 1.09	0.501	0.271	0.892
ARA	6.25 ± 0.18	5.83 ± 0.18	5.76 ± 0.44	5.37 ± 0.35	0.031	0.236	0.373
DGLA	1.10 ± 0.027	1.19 ± 0.034	0.95 ± 0.049	1.03 ± 0.046	0.023	0.353*	0.018
EDA	0.13 ± 0.003	0.15 ± 0.003	0.11 ± 0.007	0.14 ± 0.009	<0.001	0.011	0.093
DPAn-6	0.063 ± 0.004	0.068 ± 0.004	0.036 ± 0.004	0.044 ± 0.006	0.025*	0.126	<0.001*
AdA	0.11 ± 0.003	0.14 ± 0.016	0.09 ± 0.005	0.10 ± 0.008	0.004*	0.808*	0.016*
**Total PUFAn-6**	**30.3 ± 0.37**	**30.3 ± 0.41**	**29.8 ± 0.99**	**28.5 ± 1.24**	**0.965**	**0.150**	**0.653**
18:1/18:0 (DI)	5.04 ± 0.09	4.73 ± 0.08	4.78 ± 0.13	4.72 ± 0.17	0.018*	0.663	0.355
n6/n3	12.4 ± 0.41	13.6 ± 0.48	13.5 ± 0.93	12.3 ± 0.59	0.002	0.199	0.380

*AdA* adrenic acid (22:4 n-6), *ALA* α-linolenic acid (18:3 n-3), *ARA* arachidonic acid (20:4 n-6), *BCFA* branched-chain fatty acids, *CPOA2H* Cyclopropaneoctanoic A2-hexyl, *DGLA* dihomo- γ -linolenic acid (20:3 n-6), *DHA* docosahexaenoic acid (22:6 n-3), *DI* desaturation index, *DPAn*-*3* docosapentaenoic acid (22:5 n-3), *DPAn-6* docosapentaenoic acid (22:5 n-6), *ECFA* even-chain fatty acids, *EDA* eicosadienoic acid (20:2 n-6), *EPA* eicosapentaenoic acid (20:5 n-3), *LA* linoleic acid (18:2 n-6), *MUFA* monounsaturated fatty acids, *OCFA* odd-chain fatty acids, *PUFA* polyunsaturated fatty acids, *SFA* saturated fatty acids. Bold type represents the main groups of fatty acids. *p for non-parametric tests

Boldface - major groups of fatty acid

For a better visualization of the changes in FA profiles, we presented the % changes in their levels in Table 4. After rOAGB we observed a downward trend for medium-chain fatty acids (MCFAs) including C10:0 and C11:0 and greater decreases in C12:0 (*p*=0.009), as well as C13:0 (*p*<0.001), compared with pOAGB (Table 4). Patients in the rOAGB group had a trend towards a greater decrease in C23:0 from the VLCFA group than that in the patients qualified for pOAGB, as well as significantly greater decreases of other VLCFAs including C22:0 (*p*=0.042) and C24:0 (*p*=0.004) (Table 4).

**Table 4:** Changes in serum FA composition (%) in the study subjects

	pOAGB	rOAGB	*p-*value
C10	21.1 ± 14.4	−65.1 ± 3.32	0.071*
C12	125 ± 22.0	53.9 ± 36.9	0.009*
C14	49.3 ± 6.66	35.9 ± 11.6	0.477*
C16	−2.25 ± 0.83	−0.94 ± 1.32	0.301*
C18	7.37 ± 1.51	4.07 ± 1.95	0.457*
C20	11.1 ± 3.43	−1.00 ± 9.46	0.077*
C22	−10.9 ± 3.82	−18.1 ± 11.03	0.042*
C24	−5.31 ± 4.27	−25.3 ± 7.79	0.004*
**ECFA**	**0.94 ± 0.80**	**0.38 ± 1.33**	0.778*
C11	15.3 ± 12.1	−9.40 ± 30.3	0.123*
C13	28.8 ± 8.84	−53.3 ± 3.08	<0.001*
C15	20.9 ± 4.17	9.14 ± 6.55	0.131*
C17	6.91 ± 2.12	3.60 ± 7.10	0.131*
C19	40.1 ± 14.4	49.3 ± 22.9	0.575*
C21	31.1 ± 21.1	39.1 ± 52.2	0.284*
C23	−26.1 ± 4.13	−37.8 ± 10.8	0.075*
**OCFA**	**7.00 ± 2.47**	**−1.12 ± 4.21**	0.071*
6,10,12-methyl-12:0	159 ± 24.8	177 ± 27.8	0.344*
anteiso 12-M-14:0	117 ± 17.1	55.0 ± 18.5	0.094*
anteiso 14-M-16:0	56.1 ± 8.81	56.2 ± 8.76	0.598*
anteiso 16-M-18:0	63.6 ± 9.62	68.0 ± 13.5	0.545*
anteiso 21-M-22:0	66.9 ± 13.8	15.8 ± 31.4	0.352*
**anteiso BCFA**	**50.0 ± 6.25**	**35.1 ± 5.72**	0.090
iso 12-M-13:0	102 ± 23.4	26.4 ± 32.9	0.159*
iso 13-M-14:0	89.1 ± 11.9	40.7 ± 14.7	0.093*
iso 14-M-15:0	59.2 ± 6.45	56.2 ± 7.65	0.925*
iso 15-M-16:0	26.3 ± 4.75	37.6 ± 15.3	0.709*
iso 20-M-21:0	54.7 ± 14.2	103 ± 37.5	0.637*
**Iso BCFA**	**32.2 ± 3.88**	**26.5 ± 8.18**	0.520
**Total BCFA**	**39.2 ± 4.49**	**29.4 ± 4.90**	0.309
**Total SFA**	**0.87 ± 0.71**	**0.65 ± 1.29**	0.858*
C14:1	63.9 ± 13.8	41.7 ± 18.3	0.900*
C16:1	0.71 ± 2.51	18.1 ± 8.67	0.072*
C18:1	0.43 ± 1.25	2.31 ± 2.36	0.509
C19:1	39.0 ± 10.4	23.8 ± 22.3	0.438*
C20:1	3.61 ± 3.73	−20.2 ± 4.60	0.004
C22:1	34.3 ± 11.1	−1.89 ± 18.1	0.248*
C24:1	36.3 ± 4.90	47.9 ± 8.82	0.286*
**MUFA**	**0.63 ± 1.22**	**3.69 ± 2.51**	0.301
CPOA2H	10.2 ± 4.9	2.56 ± 7.56	0.592*
ALA	12.9 ± 6.67	37.4 ± 17.7	0.331*
EPA	−6.10 ± 4.54	10.1 ± 10.9	0.186
ETA	−15.6 ± 5.02	−5.79 ± 12.4	0.533*
DHA	−10.7 ± 2.86	0.31 ± 7.12	0.181
DPAn-3	14.0 ± 3.24	21.2 ± 9.67	0.915*
**n3 PUFA**	**−7.09 ± 2.58**	**3.47 ± 5.50**	0.096
LA	1.95 ± 1.78	−3.18 ± 3.95	0.249
ARA	−3.20 ± 3.23	−2.82 ± 5.04	0.598*
DGLA	12.2 ± 4.01	14.1 ± 8.87	0.968*
EDA	27.9 ± 3.81	38.4 ± 11.3	0.391
DPAn6	23.3 ± 6.55	47.3 ± 21.5	0.606*
AdA	50.4 ± 29.8	4.07 ± 9.26	0.151*
**n6 PUFA**	**0.67 ± 1.61**	**−4.24 ± 2.94**	0.157
DI (18:1/18:0)	−4.65 ± 1.94	−0.82 ± 2.55	0.242
n6/n3	10.80 ± 2.92	−2.82 ± 6.23	0.044*

*AdA* adrenic acid (22:4 n-6), *ALA* α-linolenic acid (18:3 n-3), *ARA* arachidonic acid (20:4 n-6), *BCFA* branched-chain fatty acids, *CPOA2H* Cyclopropaneoctanoic A2-hexyl, *DGLA* dihomo- γ -linolenic acid (20:3 n-6), *DHA* docosahexaenoic acid (22:6 n-3), *DI* desaturation index, *DPAn*-*3* docosapentaenoic acid (22:5 n-3), *DPAn*-*6* docosapentaenoic acid (22:5 n-6), *ECFA* even-chain fatty acids, *EDA* eicosadienoic acid (20:2 n-6), *EPA* eicosapentaenoic acid (20:5 n-3), *LA* linoleic acid (18:2 n-6), *MUFA* monounsaturated fatty acids, *OCFA* odd-chain fatty acids, *PUFA* polyunsaturated fatty acids, *SFA* saturated fatty acids. Bold type represents the main groups of fatty acids. *p for non-parametric tests

Boldface - major groups of fatty acid

BCFA levels before pOAGB and before rOAGB were not significantly different between both groups (*p*=0.463, Table 4), and after OAGB (both primary and revisional), an improvement in BCFA concentrations was observed (pOAGB 0.29 ± 0.01 vs 0.39 ± 0.01 and rOAGB 0.29 ± 0.01 vs 0.38 ± 0.02; *p*<0.001, *p*<0.001, respectively) (Table 3). In contrast to the lack of effect of rOAGB on most FAs from the PUFA group, in the pOAGB group, there was a statistically significant decrease in n3 PUFAs, including eicosapentaenoic acid (EPA, 20:5n3) (*p*=0.048), eicosatetraenoic acid (ETA, 20:4n3) (*p*<0.001), and docosahexaenoic acid (DHA, 22:6n3) (*p*<0.001), as well as in arachidonic acid (ARA, 20:4n6) (0.031) (Table 3). These results translated into significantly (*p*=0.044) different changes in the n6/n3 PUFA ratio between both groups, and the n6/n3 PUFA ratio was increased in pOAGB (Table 3, Table 4). What is interesting, in the monounsaturated fatty acid (MUFA) group we did not observe many changes. Only the level of C20:1 decreased significantly after rOAGB (Table S1), and there is a significant difference in the changes in this FA between both variants of OAGB (Table 4). After pOAGB and rOAGB, the level of C24:1 increased significantly, and there was a significant decrease in 18:1/18:0 DI after pOAGB (Table 3).

## Discussion

Our primary aim was to evaluate the effect of pOAGB and rOAGB (after SG) on serum FA profiles, and to compare the effects of both types of BS. Primary OAGB, which is the third most common BS worldwide, is associated with a worsening of the n3 PUFA level and the n6/n3 PUFA ratio. This suggests a potential increase in inflammation and an imbalance in FA composition post-operatively. This could have implications for cardiovascular disease risk and overall health. On the other hand, rOAGB faster reduced the levels of MCFAs, C13:0 and VLCFAs, which are associated with inflammation and higher CVD risk. BCFAs, which have been found to stimulate muscle protein synthesis and have been associated with immune-modulating effects, increased after pOAGB and rOAGB. A significant decrease in 18:1/18:0 DI after pOAGB could indicate a shift in dietary patterns or nutrient intake or could relate to changes in lipid metabolism, such as altered FA synthesis or oxidation pathways. Understanding the link between SG, WR, and FA profiles can potentially help in developing strategies to optimize long-term weight management and metabolic health after BS. Significantly greater weight loss was found after pOAGB than rOAGB, even though rOAGB was already a second BS. This may agree with the reports of Almalki et al. that pOAGB is followed by faster weight loss than other BS [24]. In the recent meta-analysis by Ali et al., OAGB is associated with better %EWL in the first 5 years following surgery than SG [25]. There is an ongoing debate on protentional deficiencies resulting from rapid weight loss. A recent study by Shirazi et al. found no difference between OAGB and SG in terms of malnutrition and deficiencies [26]. However, patients living with obesity are characterized by lower initial levels of n3 PUFAs [27]. In our study, we observed a significant reduction in n3 PUFAs, especially the level of DHA, after pOAGB, which is alarming, because there is evidence that n-3 PUFA has a protective effect on the brain [28]. The n-3 series of long-chain PUFA (EPA and DHA) is associated with a decreased risk of especially fatal coronary outcomes, as well as playing a pivotal role in the immune response or allergies [29]. On the other hand, after pOAGB we found a significant decrease in the level of ARA, a precursor of eicosanoids, which are associated with inflammation [30]. Unfortunately, too much of a decrease in n3 PUFA results in an increased n6/n3 PUFA ratio after pOAGB (Table 3, Table 4). Supplementation after BS is mandatory. Lifelong multivitamin supplements are crucial in the prevention of deficiencies [31]. Routine supplementation of n3 PUFA after BS is undetermined. However, according to recent articles, PUFA supplementation should be recommended after BS [32]. A great benefit of rOAGB is the lack of an increase in the n6/n3 PUFA ratio, an effect which was observed after pOAGB. It was previously reported that within a short time (up to 6 months) of BS, the levels of n6 and n3 PUFAs could decrease [12]. However, there are no studies addressing this issue in a group of patients after conversion procedures.

Undoubtedly, the advantage of both OAGB variants was a significant decrease in VLCFAs. VLCFAs are involved in CVD pathogenesis, may modulate lipid metabolism, increase the levels of atherogenic lipoproteins, and induce systemic inflammation [33]. VLCFA levels in serum or whole blood were positively associated with coronary artery disease prevalence or IR [34]. However, there are also contrary reports suggesting that arachidic acid (20:0), behenic acid (22:0), and lignoceric acid (24:0) are associated with a lower incidence of CVD and T2DM [35]. Nonetheless, as the authors point out, the mechanisms underlying the association between VLCFAs and cardiometabolic disease are not well understood [35].

In our earlier study in 2014, Kaska et al. showed a relationship between specific FAs of serum lipids, including MCFA and serum CRP-hs levels in women with obesity [36]. A positive correlation between serum CRP-hs and specific SFAs and MUFAs or a negative correlation with PUFAs decreased with the increased FA chain length. The strongest positive correlation was observed for C12:0, C14:0, C41:1, and C16:1 [36]. A significant increase in OCFAs, mainly C15:0 and C17:0, was observed only after pOAGB. C15:0 and C17:0 are known for their health benefits as they present anti-inflammatory and antioxidant properties [37]. In our current study, we also observed an increase in the concentration of BCFAs following both rOAGB and pOAGB, which was similar in both variants (Table 4). According to recent studies, BCFAs induce apoptosis in human breast cancer cells and inhibit tumor growth in cultured cells and in a mouse model [38, 39]. It was found that BCFAs may have a beneficial effect on proper gut function, and inverse correlations with inflammation, dyslipidemia, and IR in subjects with obesity [40]. This may speed up recovery from obesity-related disorders.

Our observation that after pOAGB, 18:1/18:0 DI was significantly lower corresponds with greater BMI reduction after pOAGB. Warensjo et al. [41] observed independent associations between desaturase activity indexes and mortality. Insulin-resistant individuals had a higher amount of adipose tissue 18:1/18:0 [42]. An improvement in IR after pOAGB was confirmed previously [15, 43]. Moreover, our earlier study showed an inverse correlation of iso BCFA with 18:1/18:0 DI, as well as with TG and insulin, in patients with obesity [40]. Interestingly, after both pOAGB and rOAGB procedures, the level of C24:1, known also as nervonic acid, increased. Similar results were observed by Lin et al. [44]. However, the level of C24:1 returned to baseline level 1 year after bariatric surgery [44]. Nervonic acid is known for promoting the repair and regeneration of tissues [45]. The current study indicates that increasing dietary C24:1 improves energy metabolism in mice and may be an effective strategy for the treatment of obesity and obesity-related complications [46].

The most important findings of this study might be summarized as follows: (1) rOAGB faster reduced levels of MCFAs, C13:0 and VLCFAs, which are associated with inflammation and higher CVD risk, (2) there is a higher risk of the worsening of the n3 PUFA level and n6/n3 PUFA ratio after pOAGB, (3) both pOAGB and rOAGB resulted in increased serum BCFA concentration, (4) there is a significant decrease in 18:1/18:0 DI after pOAGB, and (5) the FA profile before rOAGB was better than that before the procedure in the pOAGB group. The limitations of the present study include the relatively small sample size, especially in the rOAGB group; however, this was associated with the number of rOAGB procedures performed in our clinic in relation to pOAGB. Revisional case percentages are similar in another study [22].

## Conclusions

To the best of our knowledge, this is the first study to determine FA profiles in serum samples from patients after two variants of OAGB, preliminary bypass surgery and revisional OAGB following failed SG. In summary, the second surgery (rOAGB) provides much better results, but this is possibly due to the earlier BS and the faster improvement of lipid metabolism in the examined patients. Due to the large decrease in long-chain n-3 PUFAs after pOAGB, supplementation with omega 3 fatty acids in this group should be considered.

## Abbreviations

*AdA*: adrenic acid (22:4 n-6)
*ALA*: α-linolenic acid (18:3 n-3)
*ALB*: total albumin level in serum
*ARA*: arachidonic acid (20:4 n-6)
*BA*: bile acid
*BCFA*: branched-chain fatty acid
*BMI*: body mass index
*BS*: bariatric surgery
*CHOL*: total cholesterol
*CVD*: cardiovascular disease
*CPOA2H*: Cyclopropaneoctanoic A2-hexyl
*CRP-hs*: high-sensitive C-reactive protein
*DGLA*: dihomo- γ -linolenic acid (20:3 n-6)
*DHA*: docosahexaenoic acid (22:6 n-3)
*DI*: desaturation index
*DPAn-3*: docosapentaenoic acid (22:5 n-3)
*DPAn-6*: docosapentaenoic acid (22:5 n-6)
*ECFA*: even-chain fatty acid
*EDA*: eicosadienoic acid (20:2 n-6)
*EPA*: eicosapentaenoic acid (20:5 n-3)
*FFA*: free fatty acid
*GLU*: glucose
*HDL*: high-density lipoprotein
*IR*: insulin resistance
*LA*: linoleic acid (18:2 n-6)
*LDL*: low-density lipoprotein
*MCFA*: medium-chain fatty acid
*MUFA*: monounsaturated fatty acid
*OCFA*: odd-chain fatty acid
*pOAGB*: primary one anastomosis gastric bypass
*PRO*: total protein level in serum
*PUFA*: polyunsaturated fatty acid
*rOAGB*: revisional one anastomosis gastric bypass
*RYGB*: Roux-en-Y gastric bypass
*SFA*: saturated fatty acid
*SG*: sleeve gastrectomy
*T2DM*: type 2 diabetes
*TG*: triacylglycerol
